# The chromatin remodeling BAP complex limits tumor-promoting activity of the Hippo pathway effector Yki to prevent neoplastic transformation in *Drosophila* epithelia

**DOI:** 10.1242/dmm.030122

**Published:** 2017-10-01

**Authors:** Shilin Song, Héctor Herranz, Stephen M. Cohen

**Affiliations:** Department of Cellular and Molecular Medicine, University of Copenhagen, Blegdamsvej 3B, Copenhagen 2200N, Denmark

**Keywords:** SWI/SNF, Hippo pathway, Yorkie, Oncogenic cooperation, Cancer, *Drosophila*

## Abstract

Switch/sucrose non-fermentable (SWI/SNF) chromatin remodeling complexes are mutated in many human cancers. In this article, we make use of a *Drosophila* genetic model for epithelial tumor formation to explore the tumor suppressive role of SWI/SNF complex proteins. Members of the BAP complex exhibit tumor suppressor activity in tissue overexpressing the Yorkie (*Yki*) proto-oncogene, but not in tissue overexpressing epidermal growth factor receptor (EGFR). The Brahma-associated protein (BAP) complex has been reported to serve as a Yki-binding cofactor to support Yki target expression. However, we observed that depletion of BAP leads to ectopic expression of Yki targets both autonomously and non-autonomously, suggesting additional indirect effects. We provide evidence that BAP complex depletion causes upregulation of the Wingless (Wg) and Decapentaplegic (Dpp) morphogens to promote tumor formation in cooperation with Yki.

## Abstract (precis)

**Summary:** Using a *Drosophila* model of epithelial transformation, the authors identify the SWI/SNF BAP complex as an element that represses Yorkie activity and cooperates with the Yorkie protein in malignant tumor formation.

## INTRODUCTION

Tumors accumulate multiple genetic and epigenetic modifications, and mutations in epigenetic regulators are associated with several types of human cancer. Yes-associated protein (YAP) and transcriptional co-activator with PDZ-binding motif (TAZ) are the nuclear effectors of the Hippo pathway, regulate organ growth and are potent drivers of tumor formation (Harvey et al., 2013; Johnson and Halder, 2014; Piccolo et al., 2014; Yu et al., 2015). Even though activation of YAP and TAZ are widespread in human cancers, much remains to be learned about other factors that may cooperate with these oncogenes to promote malignant tumor formation.

Genes encoding Switch/sucrose non-fermentable (SWI/SNF) chromatin remodeling complex proteins are among the most commonly mutated in human cancer and have a crucial role in tumor suppression (Kadoch et al., 2013; Wilson and Roberts, 2011). However, specific mechanisms by which SWI/SNF complexes suppress tumor formation remain poorly understood. Using simple genetic tumor models provides a means to explore these mechanisms. Subunits of the SWI/SNF complex have been shown to play a tumor suppressive role in *Drosophila* (Eroglu et al., 2014; Koe et al., 2014; Xie et al., 2017).

Carcinomas originating in epithelial tissues are among the most common human cancers. The *Drosophila* imaginal discs are epithelial monolayers that proliferate actively during larval development, and have proven to be a useful model to study epithelial tumor formation (reviewed in Herranz et al., 2016). Overexpression of Yorkie (Yki), the fly ortholog of the YAP oncoprotein, leads to benign epithelial hyperplasia without driving the tissue into neoplasia (Dong et al., 2007; Herranz et al., 2012a; Huang et al., 2005). We made use of the wing imaginal disc of *Drosophila* to identify genes cooperating with Yki in malignant tumor formation.

In this study, we identify the SWI/SNF Brahma-associated protein (BAP) complex as a suppressor of Yki-induced tumor formation. Although depletion of the BAP remodeling complex has been shown to reduce expression of Yki target genes in the wing pouch (Oh et al., 2013; Zhu et al., 2015), we observed upregulation of Yki targets in other regions of the wing imaginal disc. We also provide evidence for an indirect effect of BAP complex depletion mediated by upregulation of the signaling proteins Wingless (Wg) and Decapentaplegic (Dpp). Ectopic Wg and, to a lesser extent, Dpp expression contributes to the tumor suppressive effect of the BAP complex in the context of excessive Yki activity.

## RESULTS

### Synergistic interaction between Yki and the SWI/SNF BAP complex

**Fig. 1.**
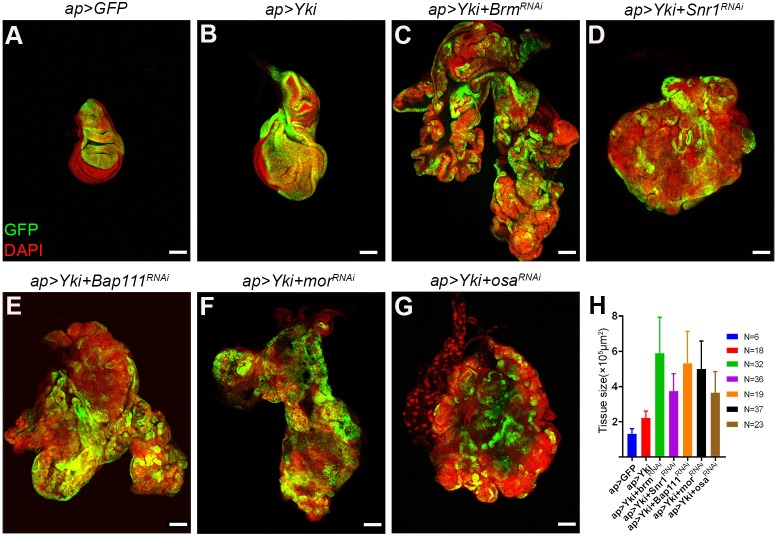
**Depletion of BAP complex subunits enhances Yki*-*induced hyperplasia.** Confocal micrographs of wing imaginal discs expressing combinations of *UAS*-transgenes under *apGal4* control. Discs were labeled with *UAS-GFP* to mark the transgene-expressing tissue (green) and with DAPI to label nuclei (red). (A) Control wing imaginal disc expressing GFP. (B) Wing imaginal disc expressing Yki. (C-G) Wing imaginal discs co-expressing *UAS-Yki* with UAS-RNAi transgenes to deplete expression of BAP complex subunit genes. Scale bars: 100 μm. (H) Quantification of tissue size (*ap>Yki* versus *ap>Yki+BAP^RNAi^s*, unpaired *t*-test, *P*<0.0001 for all).

To identify genes cooperating with Yki in malignant tumor formation, we made use of *apterous-Gal4* (*apGal4*) to direct the expression of UAS-transgenes in the dorsal compartment of the wing imaginal disc epithelium; the ventral compartment serves as an internal control (Fig. 1A). Expression of a *UAS-Yki* transgene caused overgrowth of the dorsal compartment of the disc (Fig. 1B). The larvae pupariated, but died as pupae.

To look for cooperating factors that drive neoplasia in the context of increased Yki activity, we coexpressed UAS-RNAi transgenes directed against a variety of epigenetic regulators together with *UAS-Yki*. Under these conditions, depletion of Brahma (Brm), a subunit of the SWI/SNF chromatin remodeling complex, led to massive overgrowth of the disc (Fig. 1C). Inactivating mutations in several SWI/SNF subunits have been identified at a high frequency in a variety of cancers (Wilson and Roberts, 2011). We asked whether other SWI/SNF components cooperated with Yki in the formation of tumors. *Drosophila* has two distinct SWI/SNF complexes: BAP and Polybromo-BAP (PBAP) (Mohrmann and Verrijzer, 2005; Moshkin et al., 2007). Both share a common multi-subunit core, comprising the Brm ATPase, Snr1, Bap111 and Moira (Mor) proteins. This core associates with Osa protein, to form the BAP complex, or with Polybromo, Bap170 and SAYP proteins to form the PBAP complex (Chalkley et al., 2008; Mohrmann and Verrijzer, 2005; Moshkin et al., 2007). As with Brm depletion, co-expressing *Yki* with UAS-RNAi transgenes against the other common core elements (*Snr1*, *Bap111* and *Mor*), as well as depletion of *Osa*, led to massive overgrowth (Fig. 1D-G). The resulting tissues were significantly larger than that produced by expression of *UAS-Yki* alone (Fig. 1H). At least two independent RNAi transgenes were tested for each gene, with comparable results (Table 1). Introducing UAS-RNAi transgenes targeting the PBAP-specific subunits Polybromo, Bap170 or SAYP did not lead to synergistic overgrowth when combined with *UAS-Yki* (Fig. S1). These observations suggest that the BAP SWI/SNF complex in some way limits the growth promoting effects of Yki overexpression, whereas the PBAP complex does not have this effect.

**Table 1.**
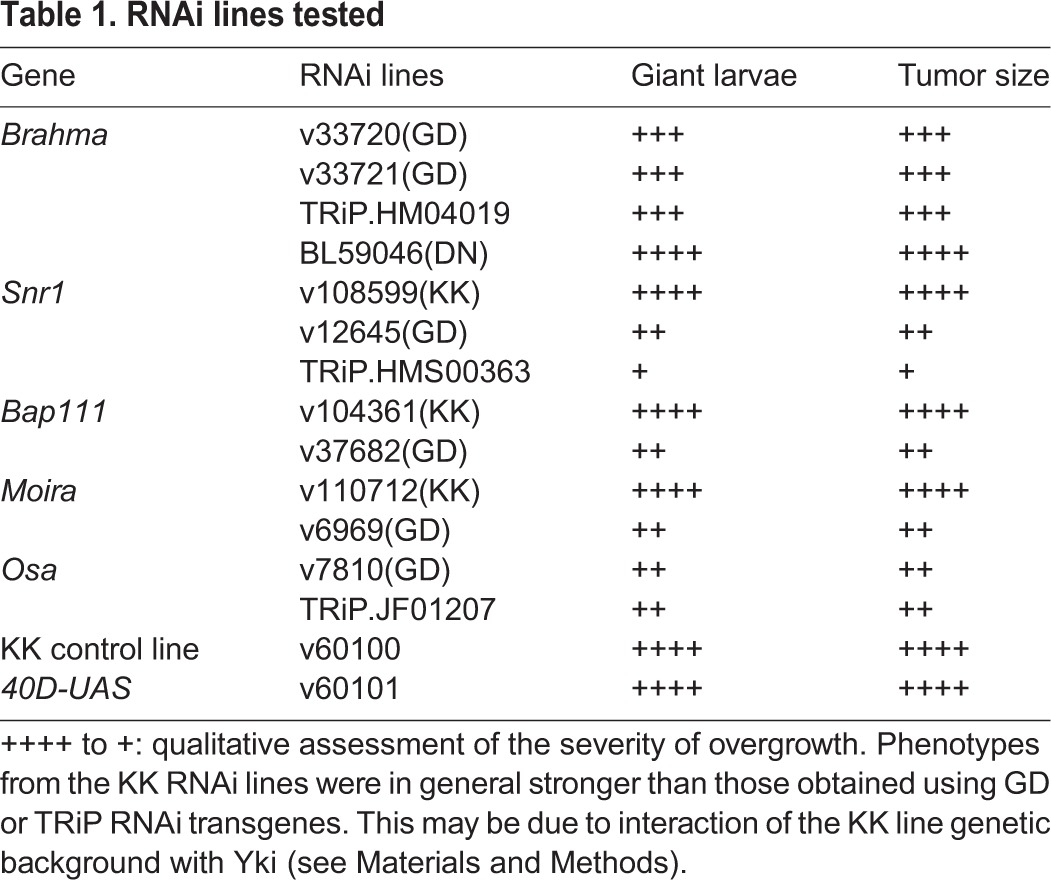
**RNAi lines tested**

### *Yki+BAP^RNAi^* tumors exhibit malignant characteristics

**Fig. 2.**
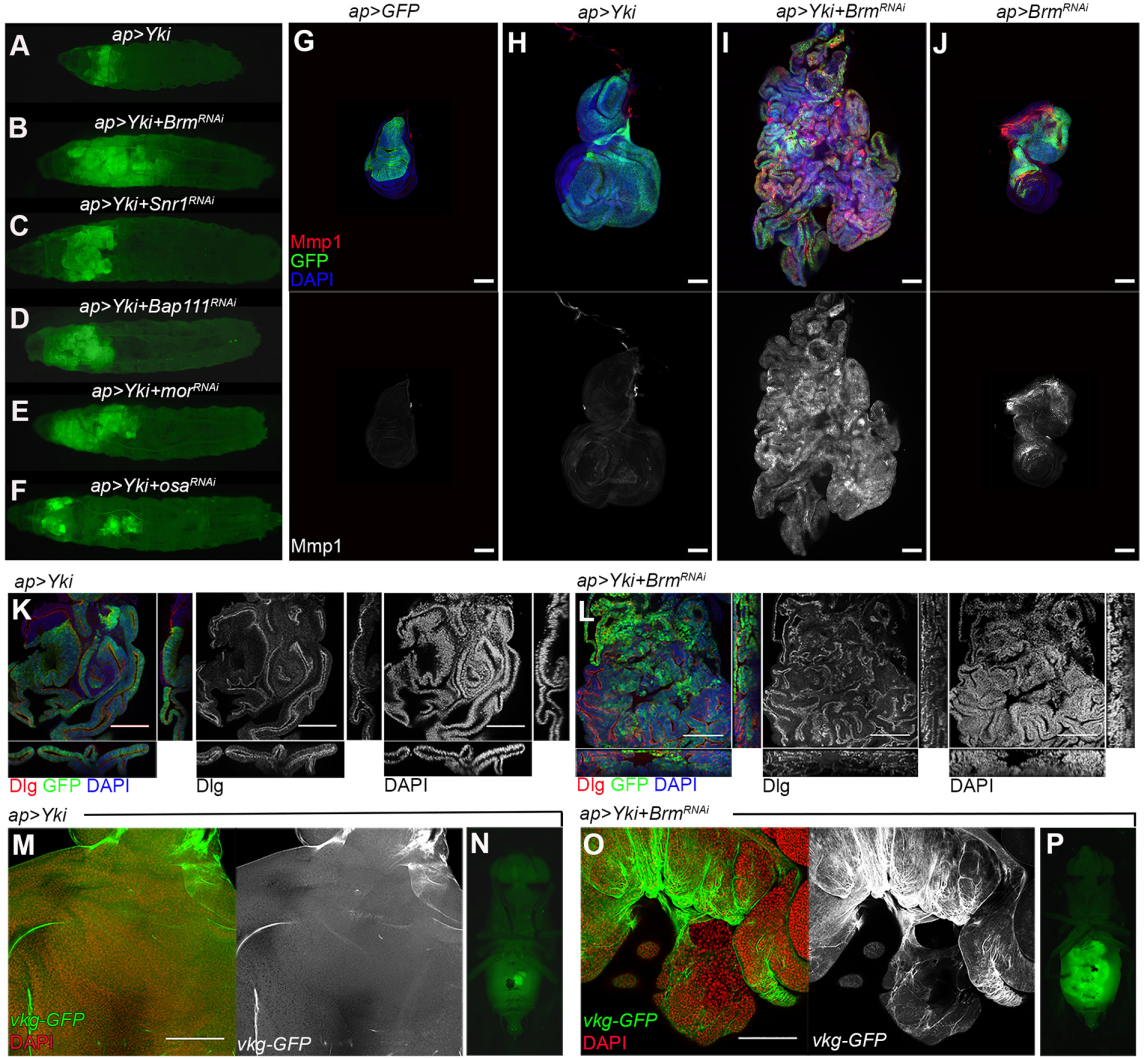
**Tumor formation by BAP-complex-depleted tissue.** (A-F) Low-magnification views of whole larvae showing the imaginal disc overgrowth for the indicated transgene combinations. GFP-expressing tissue expands massively to fill the anterior of the animal. (G-J) Confocal micrographs of wing discs expressing the indicated combinations of *UAS*-transgenes. Discs were labeled with antibody to Matrix metalloprotease 1 (Mmp1, red) as well as *UAS-GFP* to mark the Yki-expressing tissue (green) and DAPI (blue) to outline the tissue. Mmp1 channel shown separately in gray below. (K,L) Confocal micrographs of wing discs expressing the indicated combinations of *UAS*-transgenes. *xy* section is shown in the central picture; *xz* section is shown in the bottom; and *yz* section is shown in the right side. Note that the tissue in K maintains the normal epithelial organization whereas the tumor in L is highly disorganized. Discs large (Dlg) is show in red and gray. DAPI labels the nuclei and is shown in blue and gray. GFP is shown in green. (M,O) Confocal micrographs of wing discs expressing the indicated combinations of *UAS*-transgenes. *Viking* (*vkg*)-*GFP* was used to label the basement membrane and is shown in green and gray. The basement membrane appears as a continuous layer in the disc in M, whereas it is more disorganized and in some parts has been degraded in the tumor in O. (N,P) Fluorescent images of whole flies, showing GFP-expressing allograft tissue in the host abdomen. Fragments of imaginal discs were implanted in the abdomen of a host female and allowed to grow for 2 weeks. (N) *apGal4, UAS-Yki+UAS-GFP*, 0/12 showed overgrowth; (P) *apGal4, UAS-Yki+UAS-Brm^RNAi^*, 16/19 showed overgrowth. Scale bars: 100 μm.

Larvae co-expressing *UAS-Yki* and UAS-RNAi transgenes targeting BAP complex subunits under the control of the *apGal4* driver carried large tumorous discs (Fig. 2A-F; we refer to these as *Yki*+*BAP^RNAi^* larvae). Some *Yki+BAP^RNAi^* larvae did not pupate and instead continued to grow to form giant larvae, in some cases growing for more than 20 days before eventually dying. The ‘giant larva’ phenotype is characteristic of larvae with malignant tumors (Bilder, 2004). In some cases, we observed GFP-expressing tissue growths at a distance from the main tumorous mass (Fig. 2F). Ectopically located GFP-expressing tissue was never observed in larvae overexpressing Yki on its own (Fig. 2A). These ectopic GFP masses are likely to reflect metastasis from the overgrown tumorous disc.

Malignant fly tumors express the secreted matrix metalloproteinase 1 (Mmp1). Mmp1 degrades the basement membrane of the imaginal disc, allowing tumor cell migration and invasion (Beaucher et al., 2007; Uhlirova and Bohmann, 2006). In normal discs, Mmp1 is expressed in the trachea but is not detected in the proliferating epithelium (Fig. 2G). We did not detect Mmp1 expression in discs expressing *UAS-Yki* (Fig. 2H), but the *Yki+BAP^RNAi^* tumors had high levels of Mmp1 (Fig. 2I and Fig. S2A-D). Mmp1 expression correlated with degradation of basement membranes, visualized using *Viking-GFP* (*vkg-GFP*) (Fig. 2O; compare with the continuous basement membrane layer in *Yki* control discs, Fig. 2M). Interestingly, Mmp1 expression was observed when BAP complex transcripts were depleted in the absence of Yki overexpression (Fig. 2J and Fig. S2E-H). Carcinomas show defects in polarity as they evolve towards malignancy. Yki expression results in tissue overgrowth but those discs maintained normal epithelial polarity, as shown by localized expression of the apical polarity marker Discs large (Dlg, Fig. 2K). In contrast, epithelial polarity was disrupted in the *Yki+BAP^RNAi^* tumors (Fig. 2L).

Transplantation of imaginal disc fragments into the abdomen of adult hosts provides an *in vivo* assay system for tumor formation (Caussinus and Gonzalez, 2005). To assess the growth potential of the *Yki+BAP^RNAi^* tumors, we injected fragments of Yki+GFP-expressing discs, and *Yki+Brm^RNAi^* discs. Discs fragments expressing Yki alone with GFP survived in the abdomen of adult hosts 2 weeks after tumor injection, but they did not grow to form tumors (Fig. 2N). In contrast, fragments of *Yki+Brm^RNAi^* discs grew rapidly to fill much of the host abdomen and killed the hosts by 2 weeks (Fig. 2P). Taken together, these observations suggest that the *Yki+BAP^RNAi^* tumors have acquired malignant features.

### BAP complex depletion does not synergize with EGFR

**Fig. 3.**
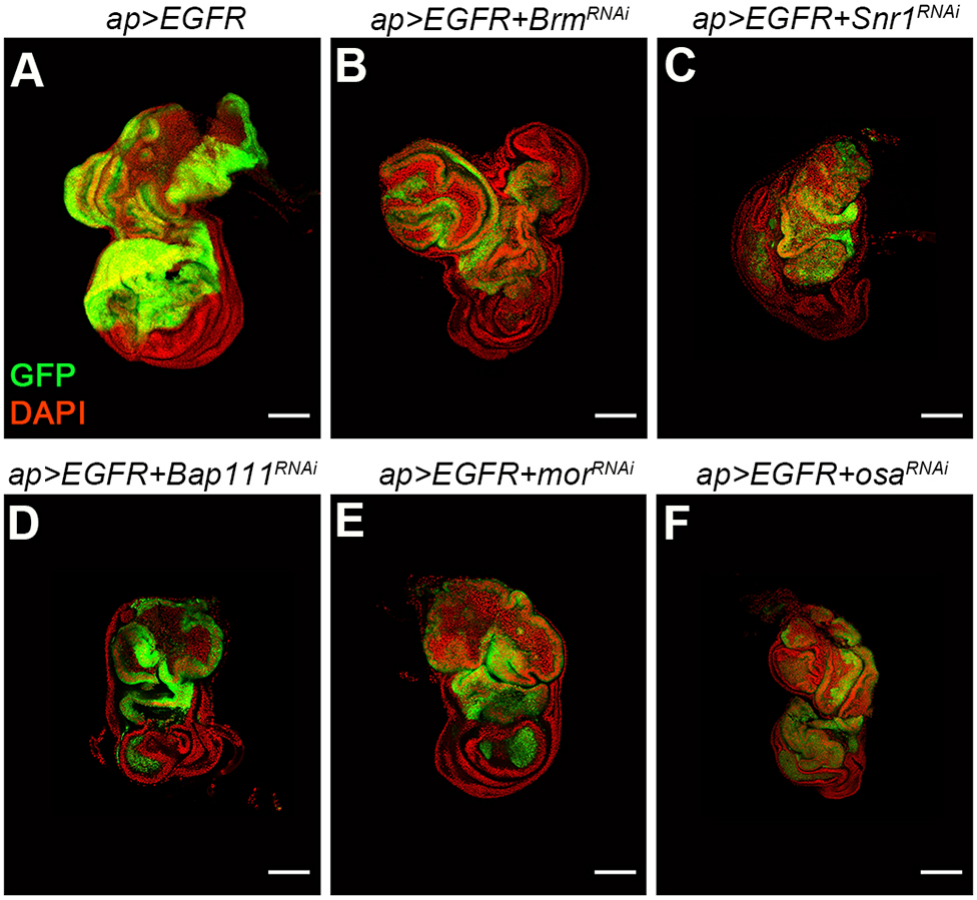
**Depletion of BAP complex subunits does not enhance EGFR*-*induced hyperplasia.** Confocal micrographs of wing discs expressing the indicated combinations of *UAS*-transgenes. Discs were labeled with *UAS-GFP* to mark the transgene-expressing tissue (green) and DAPI (red) to outline the tissue. Scale bars: 100 μm.

Activating mutations in epidermal growth factor receptors (EGFRs) and downstream effectors in the Ras/MAPK pathway are frequent in human cancer (Kandoth et al., 2013). *EGFR* behaves as an oncogene in *Drosophila*, and EGFR overexpression results in activation of the Ras/MAPK pathway and tissue hyperplasia (Herranz et al., 2012a). EGFR expression results in benign tissue overgrowth, resembling that produced by Yki overexpression (Fig. S3). As with Yki, combining EGFR with other factors can drive tumor formation (Eichenlaub et al., 2016; Herranz et al., 2012b, 2014). However, depletion of the BAP complex transcripts did not show synergistic interaction with EGFR. Growth of the tissue was similar to that produced by EGFR alone (Fig. 3A-F). These observations suggest that the BAP complex does not simply limit tissue overgrowth by any growth driver. Instead, there appears to be a specific interaction in Yki-expressing tissue.

### Depletion of BAP induces *reaper* expression and apoptosis

**Fig. 4.**
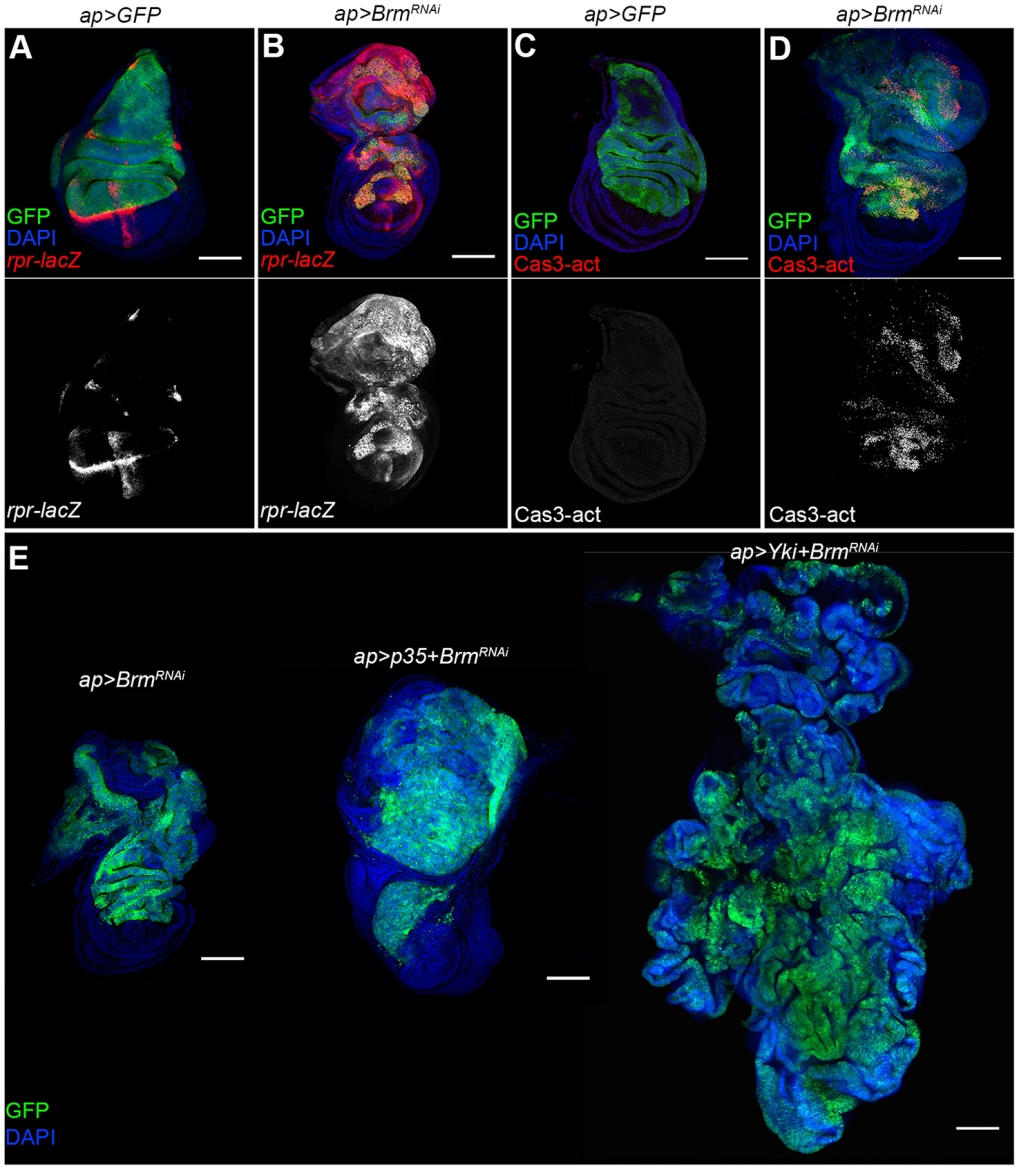
**Apoptosis in BAP-complex-depleted tissue.** Confocal micrographs of wing discs expressing the indicated combinations of *UAS*-transgenes. Discs were labeled with *UAS-GFP* to mark the transgene-expressing tissue (green) and DAPI (blue) to outline the tissue. (A,B) A *lacZ* transgene insertion at the *reaper* (*rpr*) locus was used to visualize *reaper* expression (red). (C,D) Discs were labeled with antibody to the activated form of Caspase-3 to visualize apoptotic cells (red). (E) Comparison of the effect of blocking apoptosis by expression on baculovirus p35 protein or *UAS-Yki* with *UAS-Brm^RNAi^*. Examples of other transgene combinations are shown in Fig. S4. Scale bars: 100 μm.

We next examined the consequences of depleting BAP complex subunits on their own in the developing wing epithelium. Discs depleted for different members of the BAP complex showed morphological defects and appeared moderately overgrown (Fig. 4A-D and Fig. S4A-J). These discs contained pyknotic nuclei, which are a sign of DNA fragmentation in apoptotic cells.

The *Drosophila* proapototic gene *reaper* is transcriptionally activated in response to a number of different signals (Brodsky et al., 2000; Lohmann et al., 2002; Nordstrom et al., 1996). Reaper induces apoptosis by regulating *Drosophila* Death-associated inhibitor of apoptosis protein 1 (DIAP1) (Goyal et al., 2000; Lisi et al., 2000; Yoo et al., 2002). We monitored *reaper* expression using a *lacZ* transgene inserted at the *reaper* locus. In normal discs, *reaper-lacZ* is expressed along the dorso-ventral and anterior-posterior compartment boundaries (Fig. 4A). We found that depletion of BAP components resulted in substantial ectopic *reaper-lacZ* expression (Fig. 4B and Fig. S4A-E). We also used an antibody against the activated form of Caspase-3 to visualize apoptotic cells. Apoptosis is normally undetectable in control wing discs (Fig. 4C), but many Caspase-3-positive cells were found in discs depleted of the BAP complex proteins (Fig. 4D and Fig. S4F-J).

Apoptosis is a tumor suppressor mechanism. Suppression of apoptosis can induce cell proliferation in *Drosophila* and, under some conditions, can also induce tumor formation (Huh et al., 2004; Perez-Garijo et al., 2004; Ryoo et al., 2004). To investigate whether the interaction of BAP components with Yki could be explained by suppression of apoptosis, we blocked apoptosis with the baculovirus protein p35. In discs depleted for BAP complex transcripts, co-expression of p35 led to robust overgrowth (Fig. 4E), although not as much as with the combination of p35 and *Yki+Brm^RNAi^* (Fig. 4E and Fig. S4K-O).

### BAP inhibits Yki activity in the hinge and notum regions of the wing disc

The BAP subunits Brm and Mor have been identified as Yki-associated proteins, and depletion of these subunits has been reported to downregulate Yki target genes in the wing pouch region of the wing disc (Oh et al., 2013; Zhu et al., 2015). Based on this, we should expect Brm depletion to lower Yki activity, perhaps limiting the effects of Yki-induced growth. However, we found that Brm depletion enhanced Yki-induced tissue overgrowth.

**Fig. 5.**
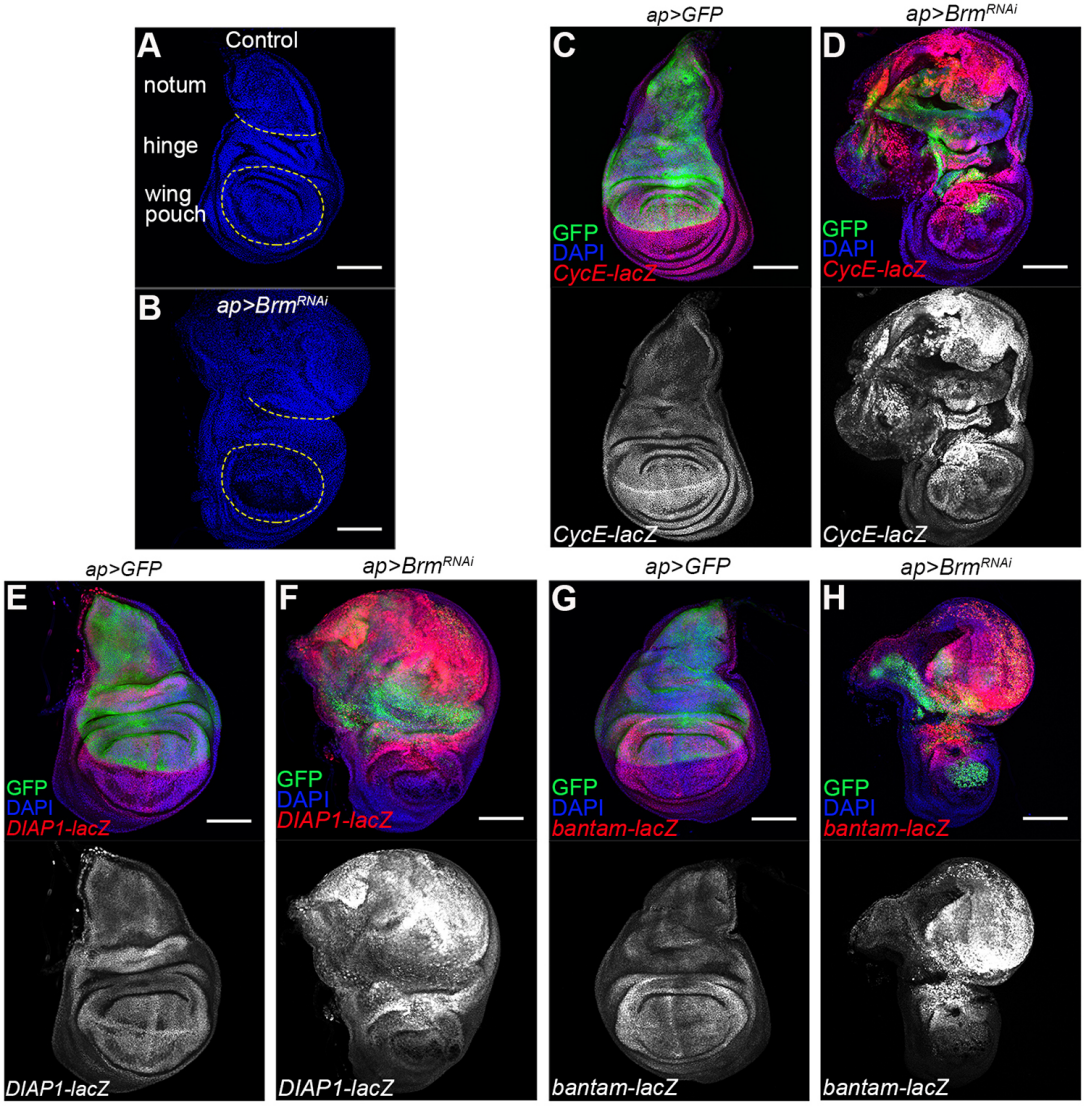
**Yki target gene expression in BAP-complex-depleted wing discs.** Confocal micrographs of wing discs expressing the indicated combinations of *UAS*-transgenes. Discs were labeled with DAPI (blue) to outline the tissue (A,B) and with *UAS-GFP* to mark the transgene-expressing tissue (green, C-H). (A,B) Schematic representation of regions of the wing disc to show that overgrowth due to *UAS-Brm^RNAi^* was mostly in the notum region. (C-H) Effects of Brm depletion on expression of Yki target genes. (C,D) *CyclinE-lacZ* transgene expression (red). (E,F) *DIAP1-lacZ* transgene expression (red). (G,H) *bantam-lacZ* transgene expression (red). Scale bars: 100 μm.

We noted that most of the tissue overgrowth in the imaginal discs depleted of the BAP complex subunits was in the region of the disc that corresponds to the wing hinge and to the presumptive dorsal thorax (notum; Fig. 5A,B), although ectopic cell death was observed throughout the *apGal4* expression domain in the dorsal wing pouch, as well as in the hinge and notum areas (Fig. 4A-D). We therefore examined expression of the Yki target genes *Cyclin E*, the inhibitor of apoptosis *DIAP1*, and *bantam* microRNA (Huang et al., 2005; Nolo et al., 2006; Thompson and Cohen, 2006). Transgenic reporters for *Cyclin E*, *DIAP1* and *bantam* showed strong ectopic expression in the overgrowing wing hinge and notum tissue in discs expressing the *UAS-BAP^RNAi^* transgenes compared to their expression levels in normal control discs, whereas expression in the wing pouch was normal to low (Fig. 5C-H and Fig. S5). Thus, the finding of BAP complex depletion causing high ectopic Yki target expression are consistent with the observed increase in tissue growth.

### Depletion of the BAP complex leads to ectopic *Wg* and *Dpp*

The observed ectopic induction of Yki targets in BAP-depleted discs would be difficult to explain solely in terms of reduced Yki activity due to depletion of the BAP cofactor proteins. This led us to consider the possibility that the BAP complex might also affect other pathways that regulate growth control in the wing disc.

**Fig. 6.**
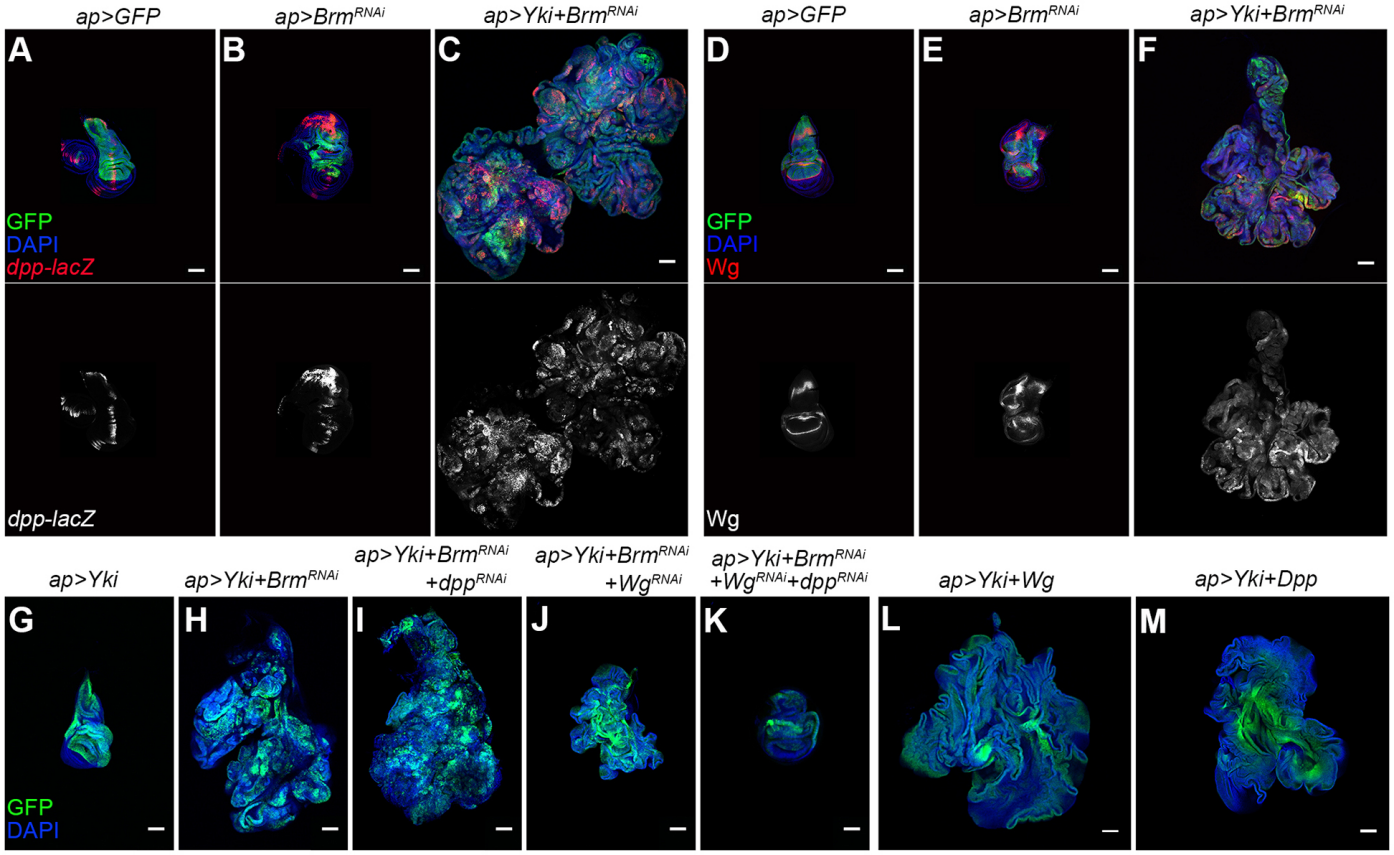
**Ectopic expression of Wg and Dpp in BAP-complex-depleted wing discs.** Confocal micrographs of wing discs expressing the indicated combinations of *UAS*-transgenes. Discs were labeled with *UAS-GFP* to mark the transgene-expressing tissue (green) and DAPI (blue) to outline the tissue. (A-C) *dpp-lacZ* was used to visualize *dpp* expression (red). (D-F) Discs were labeled with antibody to Wingless (Wg) protein (red). (G-K) Effect of RNAi-mediated depletion of *dpp* or *Wingless* in the *UAS-Yki+UAS-Brm^RNAi^* background. (L) Coexpression of *UAS-Wingless* with *UAS-Yki*. (M) Coexpression of *UAS-Dpp* with *UAS-Yki*. Scale bars: 100 μm.

The secreted growth factors Dpp and Wg are important regulators of tissue growth and cell proliferation in the wing disc (Dekanty and Milán, 2011). Using a *lacZ* reporter, we monitored *dpp* expression in discs depleted for the BAP components. *dpp-lacZ* is normally expressed in a stripe close to the anterior-posterior compartment boundary (Fig. 6A). *dpp-lacZ* was ectopically expressed in the overgrowing tissue of the BAP-complex-depleted discs (Fig. 6B and Fig. S6A-E) and was ectopically expressed throughout the *Yki+BAP^RNAi^* tumorous discs (Fig. 6C and Fig. S6F-J). Wg regulates wing disc growth and patterning, and ectopic Wg induces the formation of ectopic wing structures (Ng et al., 1996). We observed ectopic expression of Wg protein in discs depleted for the BAP complex proteins (Fig. 6D,E and Fig. S7A-E). Ectopic Wg expression was extensive in the *Yki+BAP^RNAi^* tumorous discs (Fig. 6F and Fig. S7F-J). Consistent with the earlier work of Ng et al. (Ng et al., 1996), ectopic Wg expression led to the formation of ectopic wing pouch tissue in the notum region (marked by Nubbin expression; data not shown). However, our findings contrast in part with those of Collins and Treisman (Collins and Treisman, 2000), who reported that ectopic wing tissue was formed in *osa* mutants without induction of Wg expression.

To assess the contributions of ectopic Wg and Dpp expression to tumor formation, we used RNAi-mediated depletion to limit their expression in *Yki+Brm^RNAi^* discs (Fig. 6I,J). Wg depletion strongly suppressed the growth of the *Yki+Brm^RNAi^* tumors, whereas depletion of Dpp on its own had a relatively limited effect. However, simultaneous depletion of Wg and Dpp was more effective than depletion of Wg alone (Fig. 6K), and coexpression of Yki with Wg or with Dpp proved to be sufficient to promote overgrowth (Fig. 6L,M). Thus, misexpression of Wg and Dpp each contribute to the formation of the *Yki+Brm^RNAi^* tumors.

## DISCUSSION

Benign tumors accumulate mutations that enable them to progress to malignancy and metastasis. Although Yki overexpression promotes cell proliferation and inhibits apoptosis, Yki expression does not normally lead to the formation of malignant tumors in the *Drosophila* wing epithelia. Our findings show that inactivation of the BAP complex in discs expressing Yki results in the formation of giant larvae, a phenomenon characteristic of larvae with neoplastic tumors. The overgrown imaginal discs in these animals exhibit features of malignant transformation, including loss of epithelial polarity and expression of the proinvasive marker Mmp1. Moreover, when transplanted to a normal host, fragments of these discs produced tumors that grew and spread to kill the host.

The tumor suppressive role of the BAP complex appears to be context dependent. Overexpression of EGFR and Yki each results in tissue hyperplasia. Yki regulates cell proliferation and represses apoptosis by regulating target genes, including the cell cycle regulator *Cyclin* *E*, the inhibitor of apoptosis *DIAP1* and the microRNA *bantam* (Huang et al., 2005; Nolo et al., 2006; Thompson and Cohen, 2006). Similarly, EGFR activates the Ras/MAPK pathway and induces cell proliferation by inducing Myc proto-oncogene expression (Prober and Edgar, 2000, 2002). EGFR signaling also represses apoptosis by inhibiting activity of the proapoptotic gene *Hid* (Bergmann et al., 1998; Kurada and White, 1998). Even though overexpression of EGFR and Yki resulted in a very similar growth phenotype, inactivation of BAP subunits drove tumor formation in discs expressing Yki but not in discs expressing EGFR.

Previous work has shown that some BAP subunits interact with Yki to regulate gene expression, and that Yki target gene expression was reduced following BAP complex depletion (Oh et al., 2013; Zhu et al., 2015). We were therefore surprised to find ectopic activation of Yki targets in the discs depleted for the BAP complex. Interestingly, we noted that BAP-depleted tissue was largely overgrown in the hinge and notum regions, where Yki target expression was elevated, and that the Yki targets were expressed at normal to low levels in the wing pouch in these discs (Fig. 5). Thus, there appears to be a region-specific difference in the response to BAP complex depletion. Yki regulates gene expression by interacting with a number of different DNA-binding transcription factors: Scalloped, Homothorax, Mad and Cabut (Goulev et al., 2008; Oh and Irvine, 2011; Ruiz-Romero et al., 2015; Wu et al., 2008; Zhang et al., 2008). Brm has been shown to interact with the Yki-Scalloped complex to regulate gene expression in the wing pouch (Zhu et al., 2015). Scalloped promotes wing blade development and shows high levels of expression in the wing pouch, whereas its expression levels are much lower in other regions of the wing disc (Halder et al., 1998; Simmonds et al., 1998). Homothorax is expressed in a pattern complementary to Scalloped, and acts in the hinge and notum regions of the disc (Azpiazu and Morata, 2000; Casares and Mann, 2000). It is possible that interaction of the BAP complex with the Yki-Homothorax complex might produce a different outcome with respect to Yki target gene expression than interaction with the Yki-Scalloped complex.

An alternative hypothesis is that induction of the Yki targets in the BAP-depleted notum tissue reflects an independent input. In support of this, we found that Wg and Dpp were ectopically expressed in the notum region in BAP-depleted discs as well as in the *Yki+BAP^RNAi^* discs, and that they contributed to formation of these tumors when co-expressed with Yki. Wg and Dpp are not direct targets of Yki activity but are required for normal growth of the wing imaginal disc, where they act as long-range signals to support cell survival and tissue growth. It may be of interest to explore how Wg and Dpp are ectopically induced. One possibility is that BAP complexes, acting as epigenetic factors, may normally suppress the expression of genes involved in wing pouch development in the notum region, including *Wg* and *Dpp*. This may be independent of their effects on Yki.

Another possibility involves indirect consequences of the cell death that results from BAP complex depletion. Previous reports have shown that dying cells in the wing imaginal disc produce Wg and Dpp and that blocking cell death allows for ongoing production of Wg and Dpp by the ‘undead’ cells, leading to overproliferation of the tissue (Huh et al., 2004; Perez-Garijo et al., 2004; Ryoo et al., 2004). Yki expression is anti-apoptotic, through induction of *DIAP1* and *bantam* miRNA, and we observed many cells expressing *reaper* and showing Caspase-3 activation in the *Yki+BAP^RNAi^* tumorous discs. Whereas cell death seems to predominate in the tissue depleted for the BAP complex alone (despite some induction of Wg and Dpp), co-expression with Yki leads to tissue survival and extensive overgrowth. Ultimately, this leads to acquisition of tumorous features in the tissue, including the ability to make invasive malignant tumors that can kill a host animal in allograft experiments. However, it is important to note that blocking apoptosis was not sufficient to mimic the effects of Yki expression in the BAP-complex-depleted tissue, so other Yki targets must also be important.

## MATERIALS AND METHODS

### *Drosophila* genetics

Transgenes used: *Brm^RNAi^* (v37720, v37721 and BL31712), *Snr1^RNAi^* (v12645, v108599 and BL32372), *osa^RNAi^* (v7810 and BL31266), *mor^RNAi^* (v6969 and v110712), *Bap111^RNAi^* (v38672 and v104361), *Bap170^RNAi^* (v34832), *Polybromo^RNAi^* (BL32840), *SAYP^RNAi^* (v38638), 40D-UAS (v60101), KK control (v60100), *Wg^RNAi^* (v13351), *UAS-Wg.HA* (BL5918), *UAS-dpp* (BL1486), *reaper-lacZ* (BL58793), *UAS-mCD8:GFP* (BL5137), *dpp^RNAi^* (BL25782), *UAS-p35* (BL5072), *bantam-lacZ* (P{lacW}banL1170a), *DIAP1-lacZ*, *CyclinE-lacZ*, *dpp-lacZ*, *viking-GFP* (*vkg^G454^*) (Morin et al., 2001)*.*

*UAS-GFP* was used to visualize the *apGal4*-expressing cells in all experiments. *apGal4/Gal80^ts^* was used to direct transgene expression in 3rd instar imaginal discs. Animals were reared at 18°C until early 3rd instar, and shifted to 29°C to induce transgene expression as described (Herranz et al., 2012b). Stocks used for these experiments were: (1) *apGal4*, *UAS-mCD8:GFP*/*Cyo*; *tub-Gal80^ts^*/*TM6B*; (2) *apGal4*, *UAS-GFP/Cyo*; *UAS-Yki*, *tub-Gal80^ts^*; and (3) *apGal4*, *UAS-mCD8:GFP/Cyo*; *UAS-EGFR*, *tub-Gal80*^ts^.

### Interaction of KK RNAi lines with the Hippo pathway

It has been reported that ∼25% KK lines have insertions at both 30B and 40D, and that 40D insertions can affect expression of the nearby *tiptop* gene*.* This can result in false positives in screens based on sensitized Hippo pathway phenotypes (Green et al., 2014; Vissers et al., 2016)*.* Among the KK lines used here, only v110712, targeting *Bap111*, has a 40D insertion (data not show). In each case the phenotypes obtained with KK transgenes were confirmed using independent GDP element insertion library stocks or Transgenic RNAi Project (TRiP) RNAi transgene lines (Table 1).

We tested for interaction in our screen with a known *40D-UAS* integration site as well as with the background line, which contains the original landing sites used to produce the KK collection (VDRC line 60100). As expected, the *40D-UAS* transgene showed interaction with *UAS-Yki* in tumor formation. We were surprised to find that this was also true of the original targeting line (Table 1). These tumors were qualitatively different from those resulting from BAP complex depletion (not shown). The experimental results presented in the figures were obtained with GD and TRiP RNAi transgenes, to avoid any potential bias due to cooperation with the KK line genetic background. A list of transgenes used for each figure is provided in Table S1.

### Allograft transplantation

Wing disc tissue was removed from larvae in PBS. The discs were cut into small pieces using tweezers and sharp tungsten needles and transplanted by inserting glass capillary needles into the abdomens of 1-week-old *w^1118^* female virgin host flies as described previously (Herranz et al., 2012b). The allografted flies were raised at 29°C to ensure ongoing transgene induction in the implant.

### Immunostaining and imaging

Primary antibodies used were: mouse anti-Wg [1:40, Developmental Studies Hybridoma Bank (DSHB), 4D4], mouse anti-βGal (1:50, DSHB, 40-1a), mouse anti-Mmp1 (1:10, DSHB, 3A6B4/5H7B11/3B8D12 were mixed in equal amounts), mouse anti-Dlg (1:200, DSHB, 4F3) and rabbit anti-cleaved Caspase-3 (1:500, Cell Signaling, 9661S). Samples were dissected in PBS, fixed in 4% formaldehyde for 20 min, washed for 3×10 min in PBX (1% Triton X-100 in PBS), blocked in PBX with 2% BSA (BBX) for 30 min, and incubated in BBX with primary antibody at 4°C overnight. Samples were then washed 4×30 min with BBX to remove primary antibody, and incubated in 200 µl BBX with 1 µl secondary antibody for 2 h. Alexa Fluor 555 series secondary antibodies from Thermo Fisher Scientific were used. After washing for 3×10 min in PBX (including DAPI for 1 wash), samples were mounted on glass slides. Images were taken with a Leica SP8 microscope. Image analysis was performed with Fiji software. Whole larva images were taken with a Leica Fluorescence Stereomicroscope.

### Tissue size quantification and statistics

Tissue size measure was performed with Fiji software. Statistics was performed with Prism software.

## Supplementary Material

Supplementary information
