# Supplementary material for: The chromatin remodeling BAP complex limits tumor-promoting activity of the Hippo pathway effector Yki to prevent neoplastic transformation in Drosophila epithelia
Source: Dis Model Mech. 2017 Oct 1;10(10):1201–9. doi: 10.1242/dmm.030122 (PMC5665456; doi:10.1242/dmm.030122)
Supplement: Supplementary information [file dmm-10-030122-s1.pdf]

Supplementary information

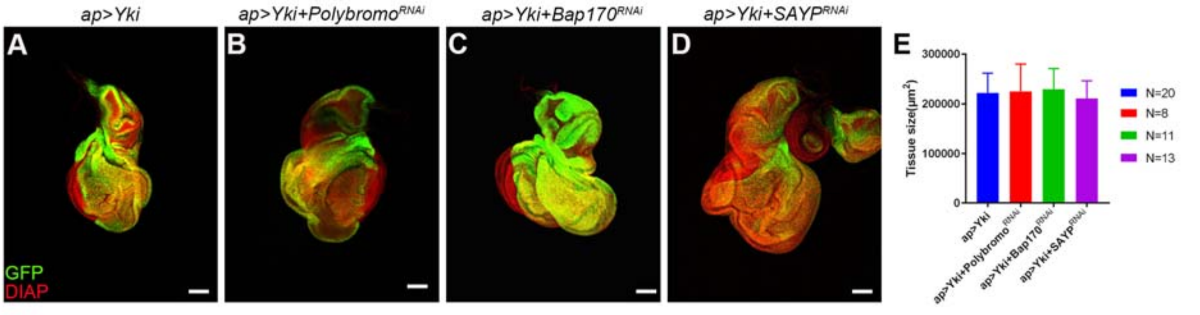

Fig. S1

Figure S1. Depletion of PBAP complex subunits does not synergize with Yki. Confocal micrographs of wing discs expressing the indicated combinations of UAS-transgenes. Discs were labeled with *UAS-GFP* to mark the transgene-expressing tissue (green) and DAPI (red) to outline the tissue (A-D). Scale bars: 100µm. (E) Quantification of tissue size (*ap>Yki* versus *ap>Yki+PBAP^{RNAi}s*, unpaired t-test: not significant).

Disease Models & Mechanisms • Supplementary information

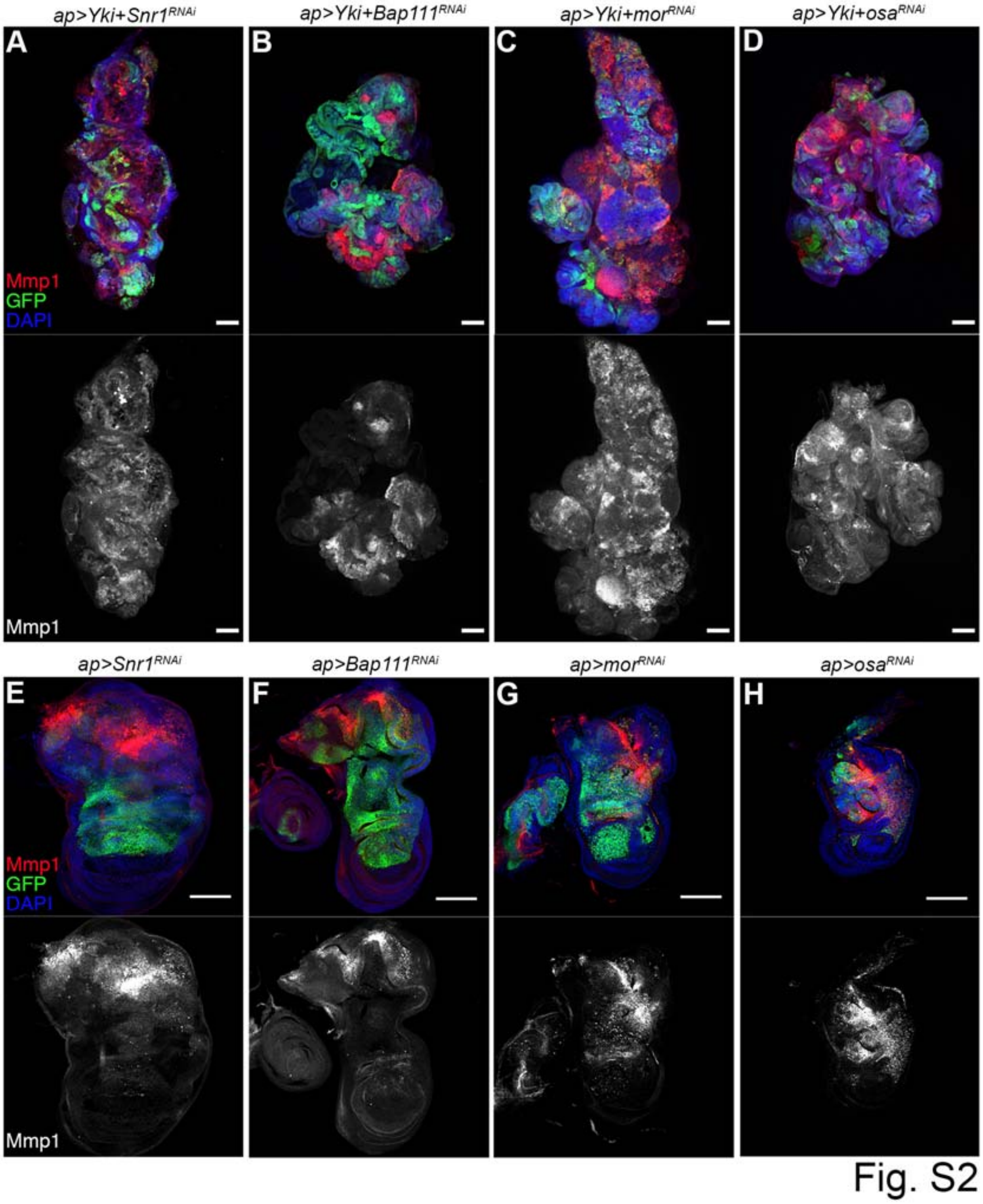

Figure S2. Mmp1 expression in BAP complex depleted discs.

Confocal micrographs of wing discs expressing the indicated combinations of UAS-transgenes. Discs were labeled with *UAS-GFP* to mark the transgene-expressing tissue (green) and DAPI (blue) to outline the tissue. Discs were labeled with antibody to Mmp1 protein (red). The Mmp1 channel is shown separately below in grey.

(A-D) discs coexpressing BAP complex UAS-RNAi transgenes together with *UAS-Yki*. (E-H) Expression of the BAP complex UAS-RNAi transgenes alone. Scale bars: 100µm.

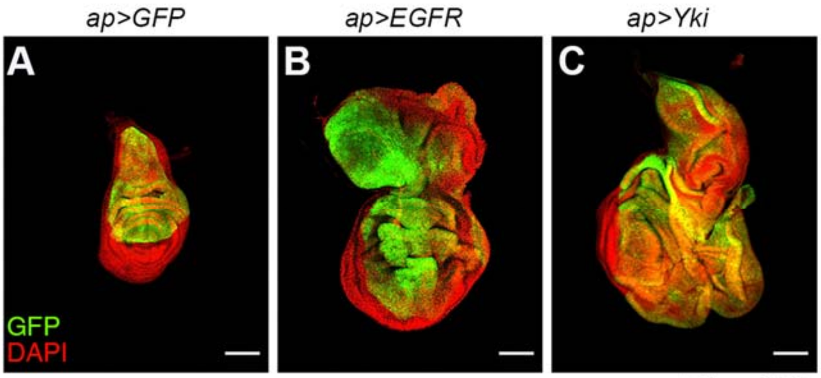

Figure S3. Tissue hyperplasia in discs overexpressing EGFR and Yki.

Confocal micrographs of wing discs expressing the indicated combinations of UAS-transgenes. Discs were labeled with *UAS-GFP* to mark the transgene-expressing tissue (green) and DAPI (red) to outline the tissue. EGFR and Yki expressing discs are similar in appearance, with the overgrown dorsal tissue thrown into folds that disturb the normally flat organization of the imaginal disc. Scale bars: 100μm.

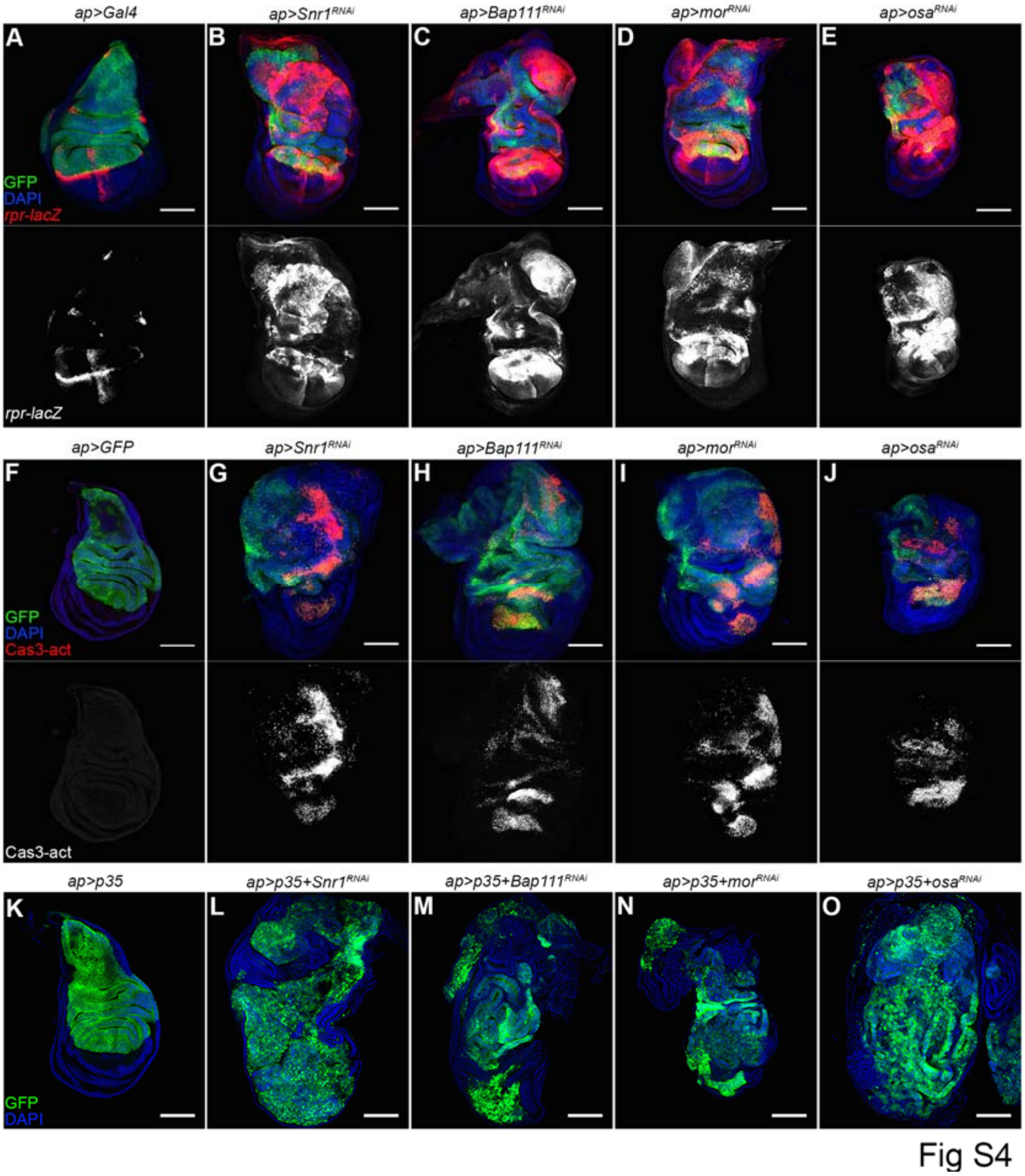

Figure S4. Apoptosis caused by BAP-complex depletion.

Confocal micrographs of wing discs expressing the indicated combinations of UAS-transgenes. Discs were labeled with *UAS-GFP* to mark the transgene-expressing tissue (green) and DAPI (blue) to outline the tissue. (A-E) *reaper-lacZ* is shown in red, and grey in the panels below. (F-J) Discs were labeled with antibody to the activated form of Caspase3 (red, grey in the panels below). (K-O) Effect of blocking apoptosis by expression of the baculovirus p35 protein in combination with BAP-complex depletion. Scale bars: 100µm.

Disease Models & Mechanisms • Supplementary information

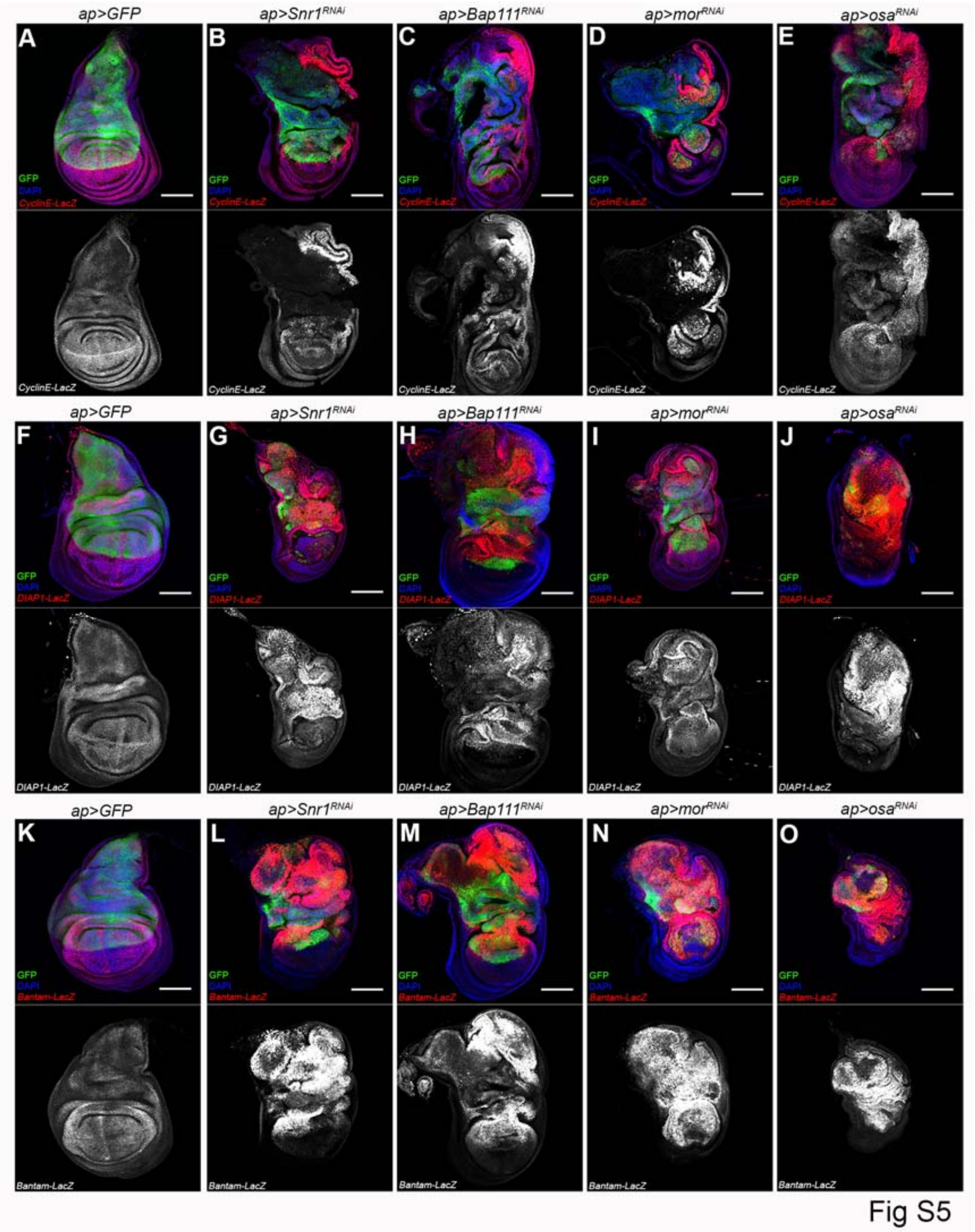

Figure S5. Effects of BAP complex depletion on expression of Yki target genes. Confocal micrographs of wing discs expressing the indicated combinations of UAS-transgenes. Discs were labeled with DAPI (blue) to outline the tissue and with *UAS-GFP* to mark the transgene-expressing tissue (green). (A-E) *CyclinE-lacZ* transgene expression (red). (F-J) *DIAP1-lacZ* transgene expression (red). (K-O) *bantam-lacZ* transgene expression (red). The LacZ channel is shown separately below in grey. Scale bars: 100µm.

Disease Models & Mechanisms • Supplementary information

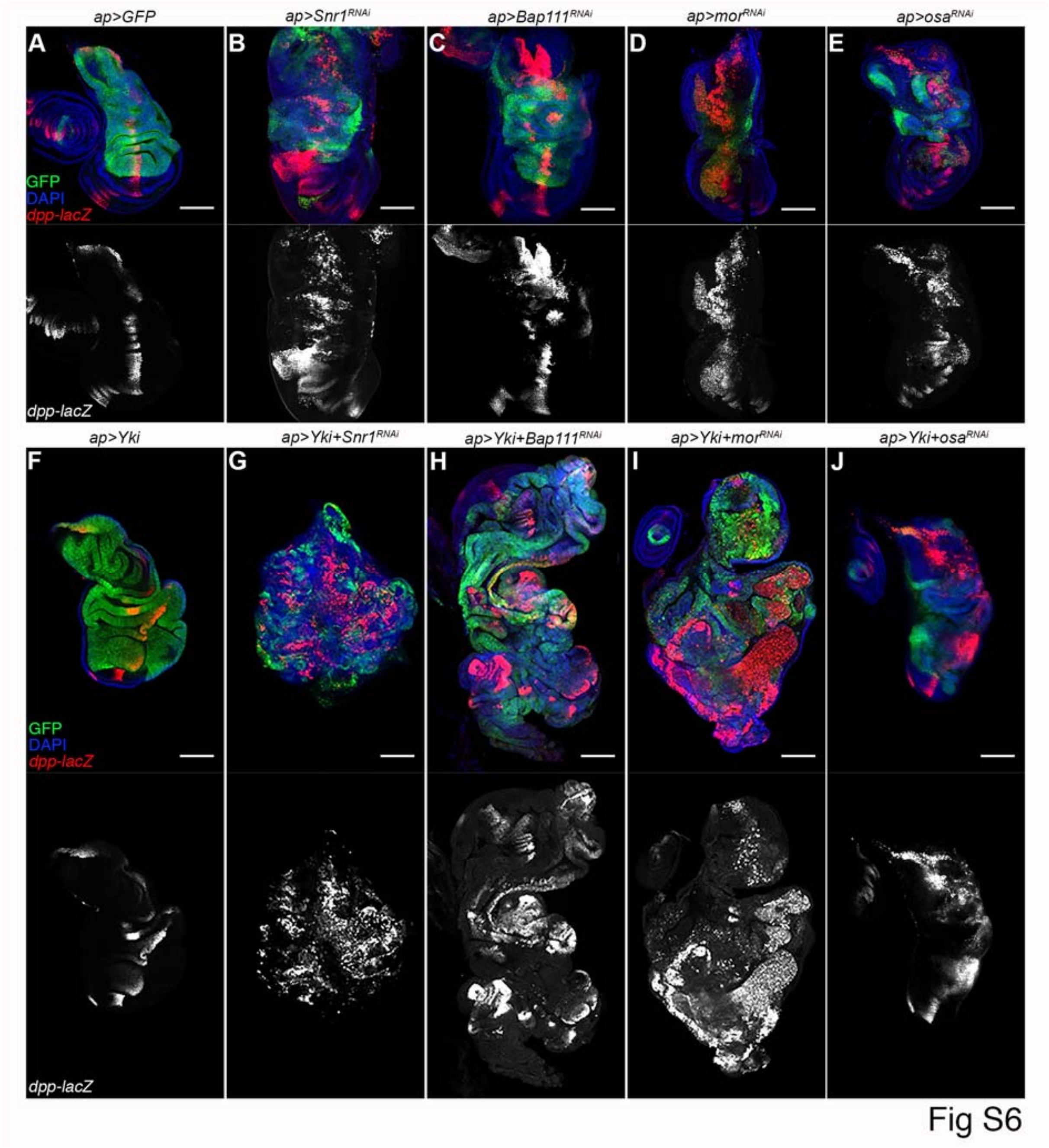

Figure S6. Dpp expression in BAP complex depleted discs.

Confocal micrographs of wing discs expressing the indicated combinations of UAS-transgenes. Discs were labeled with *UAS-GFP* to mark the transgene-expressing tissue (green) and DAPI (blue) to outline the tissue. (A-E) *dpp* expression in BAP complex depleted discs. (F-J) *dpp* expression in *Yki+BAP^{RNAi}* tumors. *dpp-lacZ* transgene expression is shown in red, and grey in the panels below. Scale bars: 100µm.

Disease Models & Mechanisms • Supplementary information

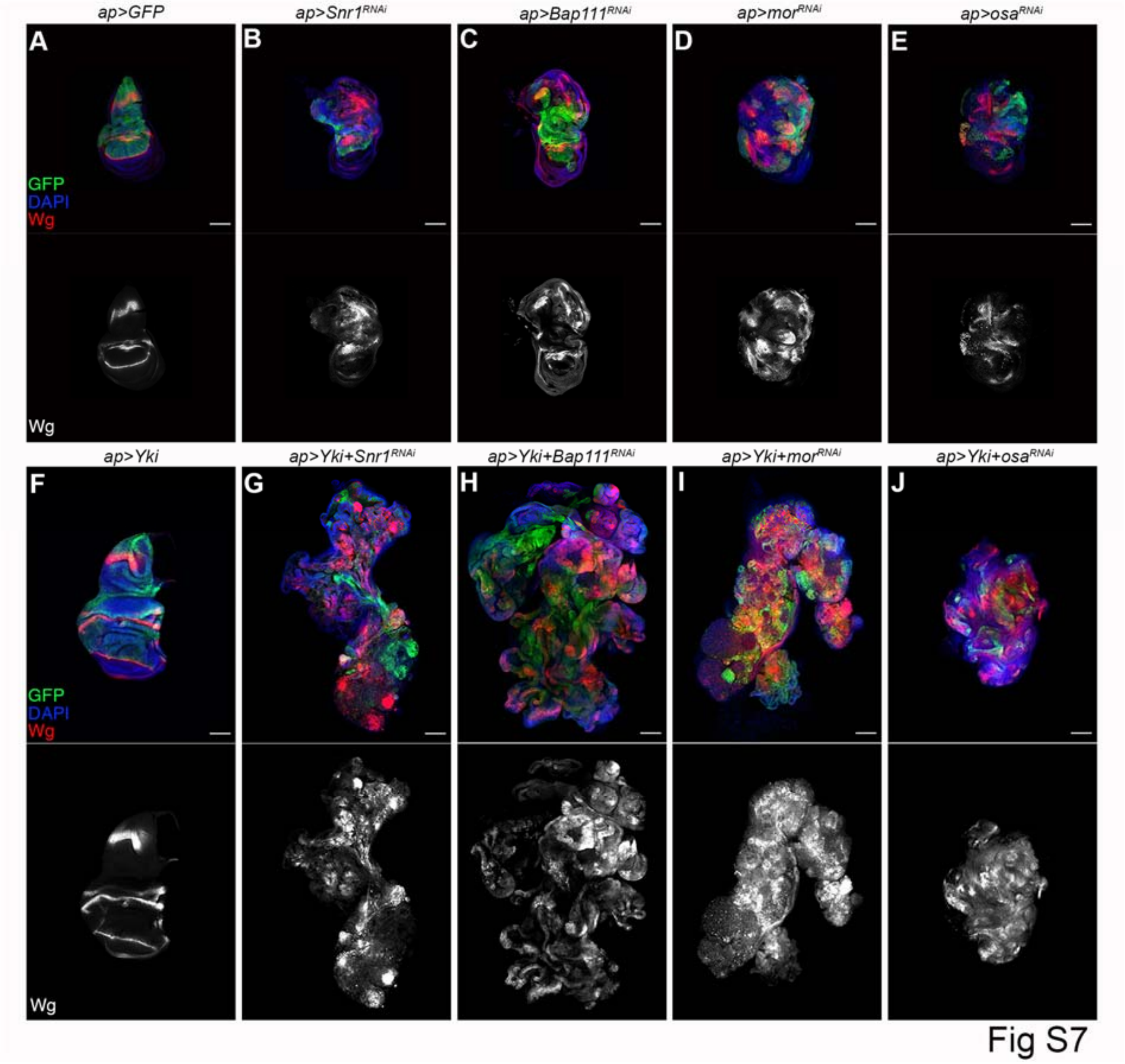

Figure S7. Wg expression in BAP complex depleted discs.

Confocal micrographs of wing discs expressing the indicated combinations of UAS-transgenes. Discs were labeled with *UAS-GFP* to mark the transgene-expressing tissue (green) and DAPI (blue) to outline the tissue. (A-E) Wg expression in BAP complex depleted discs. (F-J) Wg expression in *Yki+BAP*^{RNAi} tumors. Discs were labeled with antibody to Wg protein (red, grey in the panels below). Scale bars: 100µm.

Table S1. Detailed genotypes for each figure

Figure	Order	Genotype
Figure 1	A	apGal4, UAS-mCD8:GFP/+; tub-Gal80^{ts}/+
	B	apGal4, UAS-GFP/+; UAS-Yki, tub-Gal80^{ts}/+
	C	apGal4, UAS-GFP/+; UAS-Yki, tub-Gal80^{ts}/UAS-Brm$^{RNAi(v33720)}$
	D	apGal4, UAS-GFP/+; UAS-Yki, tub-Gal80^{ts}/UAS-Snr1$^{RNAi(v12645)}$
	E	apGal4, UAS-GFP/+; UAS-Yki, tub-Gal80^{ts}/UAS-Bap111 $^{RNAi(v37682)}$
	F	apGal4, UAS-GFP/+; UAS-Yki, tub-Gal80^{ts}/UAS-mor $^{RNAi(v6969)}$
	G	apGal4, UAS-GFP/+; UAS-Yki, tub-Gal80^{ts}/UAS-osa$^{RNAi(v7810)}$
	H	Above genotypes
Figure 2	A, H, K, M, N	apGal4, UAS-GFP/+; UAS-Yki, tub-Gal80^{ts}/+
	B, I, L, O, P	apGal4, UAS-GFP/+; UAS-Yki, tub-Gal80^{ts}/UAS-Brm$^{RNAi(v33720)}$
	C	apGal4, UAS-GFP/+; UAS-Yki, tub-Gal80^{ts}/UAS-Snr1$^{RNAi(v12645)}$
	D	apGal4, UAS-GFP/+; UAS-Yki, tub-Gal80^{ts}/UAS-Bap111 $^{RNAi(v37682)}$
	E	apGal4, UAS-GFP/+; UAS-Yki, tub-Gal80^{ts}/UAS-mor $^{RNAi(v6969)}$
	F	apGal4, UAS-GFP/+; UAS-Yki, tub-Gal80^{ts}/UAS-osa$^{RNAi(TRiP.JF01207)}$
	G	apGal4, UAS-mCD8:GFP/+; tub-Gal80^{ts}/+
	J	apGal4, UAS-mCD8:GFP/+; tub-Gal80^{ts}/ UAS-Brm$^{RNAi(v33720)}$
Figure 3	A	apGal4, UAS-mCD8:GFP/+; UAS-EGFR, tub-Gal80^{ts}/+
	B	apGal4, UAS-mCD8:GFP/+;<br>UAS-EGFR, tub-Gal80^{ts} / UAS-Brm$^{RNAi\ (v33720)}$
	C	apGal4, UAS-mCD8:GFP/+;<br>UAS-EGFR, tub-Gal80^{ts} / UAS-Snr1$^{RNAi\ (v12645)}$
	D	apGal4, UAS-mCD8:GFP/+;<br>UAS-EGFR, tub-Gal80^{ts} / UAS-Bap111$^{RNAi\ (v37682)}$
	E	apGal4, UAS-mCD8:GFP/+;<br>UAS-EGFR, tub-Gal80^{ts} / UAS-mor $^{RNAi\ (v6969)}$
	F	apGal4, UAS-mCD8:GFP/+;<br>UAS-EGFR, tub-Gal80^{ts} / UAS-osa$^{RNAi\ (v7810)}$
Figure 4	A	apGal4, UAS-mCD8:GFP/+; tub-Gal80^{ts}/reaper-LacZ
	B	apGal4, UAS-mCD8:GFP/+;<br>tub-Gal80^{ts}/reaper-LacZ, UAS-Brm$^{RNAi(v33720)}$
	C	apGal4, UAS-mCD8:GFP/+; tub-Gal80^{ts}/+
	D	apGal4, UAS-mCD8:GFP/+; tub-Gal80^{ts}/ UAS-Brm$^{RNAi(v33720)}$
	E	(left)apGal4, UAS-mCD8:GFP/+; tub-Gal80^{ts}/ UAS-Brm$^{RNAi(v33720)}$<br>(middle)apGal4, UAS-mCD8:GFP/UAS-p35; tub-Gal80^{ts}/ UAS-

		Brm$^{RNAi(v33720)}$<br>(right)*apGal4, UAS-GFP/+; UAS-Yki, tub-Gal80*^{ts}*/UAS-Brm*$^{RNAi(v33720)}$
Figure 5	A	*apGal4, UAS-mCD8:GFP/+; tub-Gal80*^{ts}*/+*
	B	*apGal4, UAS-mCD8:GFP/+; tub-Gal80*^{ts}*/ UAS-Brm*$^{RNAi(v33720)}$
	C	*apGal4, UAS-mCD8:GFP/ CyclinE-LacZ; tub-Gal80*^{ts}*/+*
	D	*apGal4, UAS-mCD8:GFP/ CyclinE-LacZ;*<br>*tub-Gal80*^{ts}*/ UAS-Brm*$^{RNAi(v33720)}$
	E	*apGal4, UAS-mCD8:GFP/+; tub-Gal80*^{ts}*/ DIAP1-LacZ*
	F	*apGal4, UAS-mCD8:GFP/+;*<br>*tub-Gal80*^{ts}*/ UAS-Brm*$^{RNAi(v33720)}$*, DIAP1-LacZ*
	G	*apGal4, UAS-mCD8:GFP/+; tub-Gal80*^{ts}*/bantam-LacZ*
	H	*apGal4, UAS-mCD8:GFP/+;*<br>*tub-Gal80*^{ts}*/ UAS-Brm*$^{RNAi(v33720)}$*, bantam-LacZ*
Figure 6	A	*apGal4, UAS-mCD8:GFP/dpp-LacZ; tub-Gal80*^{ts}*/+*
	B	*apGal4, UAS-mCD8:GFP/dpp-LacZ; tub-Gal80*^{ts}*/ UAS-Brm*$^{RNAi(v33720)}$
	C	*apGal4, UAS-GFP/ dpp-LacZ; UAS-Yki, tub-Gal80*^{ts}*/UAS-Brm*$^{RNAi(v33720)}$
	D	*apGal4, UAS-mCD8:GFP/+; tub-Gal80*^{ts}*/+*
	E	*apGal4, UAS-mCD8:GFP/+; tub-Gal80*^{ts}*/ UAS-Brm*$^{RNAi(v33720)}$
	F, H	*apGal4, UAS-GFP/+; UAS-Yki, tub-Gal80*^{ts}*/UAS-Brm*$^{RNAi(v33720)}$
	G	*apGal4, UAS-GFP/+; UAS-Yki, tub-Gal80*^{ts}*/+*
	I	*apGal4, UAS-GFP/+;*<br>*UAS-Yki, tub-Gal80*^{ts}*/UAS-dpp*$^{RNAi(BL25782,\ TRiP.JF01371)}$*, UAS-Brm*$^{RNAi(v33720)}$
	J	*apGal4, UAS-GFP/UAS-Wg*$^{RNAi(v13351)}$*;*<br>*UAS-Yki, tub-Gal80*^{ts}*/UAS-Brm*$^{RNAi(v33720)}$
	K	*apGal4, UAS-GFP/UAS-Wg*$^{RNAi(v13351)}$*; UAS-Yki, tub-Gal80*^{ts}*/ UAS-*<br>*dpp*$^{RNAi(BL25782,\ TRiP.JF01371)}$*, UAS-Brm*$^{RNAi(v33720)}$
	L	*apGal4, UAS-GFP/+; UAS-Yki, tub-Gal80*^{ts}*/UAS-Wg.HA*
	M	*apGal4, UAS-GFP/+; UAS-Yki, tub-Gal80*^{ts}*/UAS-Dpp*
Figure S1	A	*apGal4, UAS-GFP/+; UAS-Yki, tub-Gal80*^{ts}*/+*
	B	*apGal4, UAS-GFP/+; UAS-Yki, tub-Gal80*^{ts}*/UAS-Polybromo*$^{RNAi(BL32840)}$
	C	*apGal4, UAS-GFP/+; UAS-Yki, tub-Gal80*^{ts}*/ UAS-Bap170*$^{RNAi(v34582)}$
	D	*apGal4, UAS-GFP/+; UAS-Yki, tub-Gal80*^{ts}*/ SAYP*$^{RNAi(v38638)}$
	E	*Above genotypes*
Figure S2	A	*apGal4, UAS-GFP/+; UAS-Yki, tub-Gal80*^{ts}*/ UAS-Snr1*$^{RNAi(v12645)}$
	B	*apGal4, UAS-GFP/+; UAS-Yki, tub-Gal80*^{ts}*/UAS-Bap111* $^{RNAi(v37682)}$
	C	*apGal4, UAS-GFP/+; UAS-Yki, tub-Gal80*^{ts}*/UAS-mor* $^{RNAi(v6969)}$
	D	*apGal4, UAS-GFP/+; UAS-Yki, tub-Gal80*^{ts}*/UAS-osa*$^{RNAi(v7810)}$

Disease Models & Mechanisms • Supplementary information

	E	*apGal4, UAS-mCD8:GFP/+; tub-Gal80^{ts}/UAS-Snr1$^{RNAi(v12645)}$*
	F	*apGal4, UAS-mCD8:GFP/+; tub-Gal80^{ts}/UAS-Bap111 $^{RNAi(v37682)}$*
	G	*apGal4, UAS-mCD8:GFP/+; tub-Gal80^{ts}/UAS-mor $^{RNAi(v6969)}$*
	H	*apGal4, UAS-mCD8:GFP/+; tub-Gal80^{ts}/UAS-osa$^{RNAi(v7810)}$*
Figure S3	A	*apGal4, UAS-mCD8:GFP/+; tub-Gal80^{ts}/+*
	B	*apGal4, UAS-mCD8:GFP/+; UAS-EGFR, tub-Gal80^{ts}/+*
	C	*apGal4, UAS-GFP/+; UAS-Yki, tub-Gal80^{ts}/+*
Figure S4	A	*apGal4, UAS-mCD8:GFP/+; tub-Gal80^{ts}/rpr-LacZ*
	B	*apGal4, UAS-mCD8:GFP/+; tub-Gal80^{ts}/rpr-LacZ, UAS-Snr1$^{RNAi(v12645)}$*
	C	*apGal4, UAS-mCD8:GFP/+;*<br>*tub-Gal80^{ts}/rpr-LacZ, UAS-Bap111 $^{RNAi(v37682)}$*
	D	*apGal4, UAS-mCD8:GFP/+; tub-Gal80^{ts}/rpr-LacZ, UAS-mor $^{RNAi(v6969)}$*
	E	*apGal4, UAS-mCD8:GFP/+; tub-Gal80^{ts}/rpr-LacZ, UAS-osa$^{RNAi(v7810)}$*
	F	*apGal4, UAS-mCD8:GFP/+; tub-Gal80^{ts}/ +*
	J	*apGal4, UAS-mCD8:GFP/+; tub-Gal80^{ts}/ UAS-Snr1$^{RNAi(v12645)}$*
	H	*apGal4, UAS-mCD8:GFP/+; tub-Gal80^{ts}/ UAS-Bap111 $^{RNAi(v37682)}$*
	I	*apGal4, UAS-mCD8:GFP/+; tub-Gal80^{ts}/ UAS-mor $^{RNAi(v6969)}$*
	J	*apGal4, UAS-mCD8:GFP/+; tub-Gal80^{ts}/ UAS-osa$^{RNAi(v7810)}$*
	K	*apGal4, UAS-mCD8:GFP/UAS-p35; tub-Gal80^{ts}/ +*
	L	*apGal4, UAS-mCD8:GFP/UAS-p35; tub-Gal80^{ts}/ UAS-Snr1$^{RNAi(v12645)}$*
	M	*apGal4, UAS-mCD8:GFP/UAS-p35; tub-Gal80^{ts}/ UAS-Bap111 $^{RNAi(v37682)}$*
	N	*apGal4, UAS-mCD8:GFP/UAS-p35; tub-Gal80^{ts}/ UAS-mor $^{RNAi(v6969)}$*
	O	*apGal4, UAS-mCD8:GFP/UAS-p35; tub-Gal80^{ts}/ UAS-osa$^{RNAi(v7810)}$*
Figure S5	A	*apGal4, UAS-mCD8:GFP/ CyclinE-LacZ; tub-Gal80^{ts}/+*
	B	*apGal4, UAS-mCD8:GFP/ CyclinE-LacZ;*<br>*tub-Gal80^{ts}/ UAS-Snr1$^{RNAi(v12645)}$*
	C	*apGal4, UAS-mCD8:GFP/ CyclinE-LacZ;*<br>*tub-Gal80^{ts}/ Bap111 $^{RNAi(v37682)}$*
	D	*apGal4, UAS-mCD8:GFP/ CyclinE-LacZ;*<br>*tub-Gal80^{ts}/ UAS-mor $^{RNAi(v6969)}$*
	E	*apGal4, UAS-mCD8:GFP/ CyclinE-LacZ;*<br>*tub-Gal80^{ts}/ UAS-osa$^{RNAi(v7810)}$*
	F	*apGal4, UAS-mCD8:GFP/+; tub-Gal80^{ts}/ DIAP1-LacZ*
	G	*apGal4, UAS-mCD8:GFP/+;*<br>*tub-Gal80^{ts}/ UAS-Snr1$^{RNAi(v12645)}$, DIAP1-LacZ*
	H	*apGal4, UAS-mCD8:GFP/+;*<br>*tub-Gal80^{ts}/ UAS-Bap111 $^{RNAi(v37682)}$, DIAP1-LacZ*

Disease Models & Mechanisms • Supplementary information

	I	*apGal4, UAS-mCD8:GFP/+;*<br>*tub-Gal80^{ts}/ UAS-mor$^{RNAi(v6969)}$, DIAP1-LacZ*
	J	*apGal4, UAS-mCD8:GFP/+;*<br>*tub-Gal80^{ts}/ UAS-osa$^{RNAi(v7810)}$, DIAP1-LacZ*
	K	*apGal4, UAS-mCD8:GFP/+; tub-Gal80^{ts}/bantam-LacZ*
	L	*apGal4, UAS-mCD8:GFP/+;*<br>*tub-Gal80^{ts}/ UAS-Snr1$^{RNAi(v12645)}$, bantam-LacZ*
	M	*apGal4, UAS-mCD8:GFP/+;*<br>*tub-Gal80^{ts}/ UAS-Bap111$^{RNAi(v37682)}$, bantam-LacZ*
	N	*apGal4, UAS-mCD8:GFP/+;*<br>*tub-Gal80^{ts}/ UAS-mor$^{RNAi(v6969)}$, bantam-LacZ*
	O	*apGal4, UAS-mCD8:GFP/+;*<br>*tub-Gal80^{ts}/ UAS-osa$^{RNAi(v7810)}$, bantam-LacZ*
Figure S6	A	*apGal4, UAS-mCD8:GFP/dpp-LacZ; tub-Gal80^{ts}/+*
	B	*apGal4, UAS-mCD8:GFP/dpp-LacZ; tub-Gal80^{ts}/ UAS-Snr1$^{RNAi(v12645)}$*
	C	*apGal4, UAS-mCD8:GFP/dpp-LacZ;*<br>*tub-Gal80^{ts}/ UAS-Bap111$^{RNAi(v37682)}$*
	D	*apGal4, UAS-mCD8:GFP/dpp-LacZ; tub-Gal80^{ts}/ UAS-mor$^{RNAi(v6969)}$*
	E	*apGal4, UAS-mCD8:GFP/dpp-LacZ; tub-Gal80^{ts}/ UAS-osa$^{RNAi(v7810)}$*
	F	*apGal4, UAS-GFP/ dpp-LacZ; UAS-Yki, tub-Gal80^{ts}/+*
	G	*apGal4, UAS-GFP/ dpp-LacZ; UAS-Yki, tub-Gal80^{ts}/ UAS-Snr1$^{RNAi(v12645)}$*
	H	*apGal4, UAS-GFP/ dpp-LacZ;*<br>*UAS-Yki, tub-Gal80^{ts}/ UAS-Bap111$^{RNAi(v37682)}$*
	I	*apGal4, UAS-GFP/ dpp-LacZ; UAS-Yki, tub-Gal80^{ts}/ UAS-mor$^{RNAi(v6969)}$*
	J	*apGal4, UAS-GFP/ dpp-LacZ; UAS-Yki, tub-Gal80^{ts}/ UAS-osa$^{RNAi(v7810)}$*
Figure S7	A	*apGal4, UAS-mCD8:GFP/+; tub-Gal80^{ts}/+*
	B	*apGal4, UAS-mCD8:GFP/+; tub-Gal80^{ts}/UAS-Snr1$^{RNAi(v12645)}$*
	C	*apGal4, UAS-mCD8:GFP/+; tub-Gal80^{ts}/UAS-Bap111$^{RNAi(v37682)}$*
	D	*apGal4, UAS-mCD8:GFP/+; tub-Gal80^{ts}/UAS-mor$^{RNAi(v6969)}$*
	E	*apGal4, UAS-mCD8:GFP/+; tub-Gal80^{ts}/UAS-osa$^{RNAi(v7810)}$*
	F	*apGal4, UAS-GFP/+; UAS-Yki, tub-Gal80^{ts}/+*
	G	*apGal4, UAS-GFP/+; UAS-Yki, tub-Gal80^{ts}/UAS-Snr1$^{RNAi(v12645)}$*
	H	*apGal4, UAS-GFP/+; UAS-Yki, tub-Gal80^{ts}/UAS-Bap111$^{RNAi(v37682)}$*
	I	*apGal4, UAS-GFP/+; UAS-Yki, tub-Gal80^{ts}/UAS-mor$^{RNAi(v6969)}$*
	J	*apGal4, UAS-GFP/+; UAS-Yki, tub-Gal80^{ts}/UAS-osa$^{RNAi(v7810)}$*
